# International Normalized Ratio-to-Albumin Ratio as a Novel Marker of Upper Gastrointestinal Bleeding Severity

**DOI:** 10.1155/2022/1172540

**Published:** 2022-10-13

**Authors:** Jeongwoo Choi, Je Seop Lee, Solmoon Lee, Yong Won Kim, Yoonsuk Lee, Tae Youn Kim

**Affiliations:** ^1^Department of Emergency Medicine, Yonsei University Wonju College of Medicine, Wonju, Republic of Korea; ^2^Department of Emergency Medicine, Dongguk University Ilsan Hospital, Dongguk University College of Medicine, Goyang, Republic of Korea; ^3^Research Institute of Hyperbaric Medicine and Science, Yonsei University Wonju College of Medicine, Wonju, Republic of Korea

## Abstract

**Introduction:**

Upper gastrointestinal bleeding (UGIB) is a potentially life-threatening gastrointestinal emergency, and effective management depends on early risk stratification. The Glasgow–Blatchford and Rockall scores are commonly used prognostic measures for UGIB, although these scoring systems are relatively difficult to apply in early emergency settings. AIMS65 with five items, albumin, international normalized ratio, mental status, systolic blood pressure, and age (>65 years), showed efficacy in predicting long-term hospitalization and mortality. This study aimed to investigate the usefulness of the prothrombin time-international normalized ratio-to-albumin ratio (PTAR) in the emergency room for early UGIB risk stratification.

**Methods:**

We retrospectively examined patients who visited a tertiary academic hospital's emergency department (ED) with UGIB as the chief presentation between January 2019 and December 2020. The cutoff values and diagnostic accuracies of the PTAR, Glasgow–Blatchford score, AIMS65 score, pre-endoscopy, and complete Rockall score were analyzed, and the performance of the PTAR was compared with that of other risk stratification methods. In total, 519 patients were enrolled: 163 patients were admitted in the intensive care unit (ICU) and 35 died during admission. Multiple logistic regression analyses confirmed the association of the PTAR with ICU admission and mortality. The adjusted odd ratio (aOR) of the PTAR for ICU admission care was 8.376 (2.722–25.774), and the aOR of the PTAR for mortality was 27.846 (8.701–89.116).

**Conclusions:**

The PTAR measured in the ED is an independent factor related to ICU admission and mortality in patients with UGIB. Using ED blood laboratory results, which are reported relatively quickly and are easy to acquire and calculate, the PTAR can be used as a risk stratification marker in the early emergency setting.

## 1. Introduction

Upper gastrointestinal bleeding (UGIB) requires frequent hospitalizations and carries substantial morbidity and mortality risks. In the United States, UGIB incurs over $1 billion in direct medical costs annually [1, 2]. UGIB is a potentially life-threatening gastrointestinal emergency, and early risk stratification can be useful to determine effective management [3]. The Glasgow–Blatchford Score (GBS), Rockall score, and albumin, international normalized ratio, mental status, systolic blood pressure, age>65 years (AIMS65) score are prognostic tools for UGIB [4]. However, they are challenging to apply in the emergency setting, as they require various laboratory and clinical variables, including endoscopy, to predict prognosis [5].

Laboratory tests for UGIB have been used in various ways to predict prognosis [6, 7]. In particular, complete blood count and chemistry results have been used for early prognostic prediction in the emergency department (ED) [8, 9]. A new objective liver function score model called the prothrombin time-international normalized ratio (PT-INR)-to-albumin ratio (PTAR) has been used for risk stratification in hepatic cellular carcinoma and sepsis [8, 10]. Decreased liver function is associated with mortality and morbidity in patients with UGIB [11]; thus, it is recommended to measure liver function in the ED [12]. The PTAR may be a potentially useful marker for assessing liver function in patients with UGIB. This study aimed to investigate the usefulness of the PTAR measured in the ED and compare it to other risk stratification methods (e.g., AIMS65, Rockall score, and GBS) for UGIB.

## 2. Materials and Methods

### 2.1. Study Design and Participants

This retrospective observational study was performed at the ED of a tertiary university hospital which accommodates 43,000 patients annually, approximately 250 of whom present with UGIB. This study was approved by the Institutional Review Board of Wonju Severance Christian Hospital (IRB No. CR321093). The study protocol conformed to the ethical guidelines of the 1975 Declaration of Helsinki, and the requirement for written informed consent was waived owing to the retrospective nature of the study.

In total, 520 patients, who visited the ED with UGIB as the chief presentation between January 2019 and December 2020, were evaluated. Patients with UGIB as a discharge code in the computerized hospital records were eligible for inclusion in this study. The exclusion criteria were age <19 years.

### 2.2. Study Variables

Data were retrospectively collected from the electronic medical records. These included age, sex, intensive care unit (ICU) admission, and mortality. Systolic blood pressure, pulse rate, hemoglobin, platelet count, blood urea nitrogen, creatinine, PT-INR, and albumin levels measured within 1 hour of ED arrival were also analyzed. Comorbidities include hypertension, diabetes mellitus, liver cirrhosis, chronic renal failure, and heart failure. The PTAR was measured in the ED using serum laboratory parameters. Both the pre-endoscopy and complete Rockall scores were retrospectively calculated. The pre-endoscopy Rockall score is calculated using age; presence of shock; presence of comorbidities such as cardiac failure, ischemic heart disease, renal, or liver failure; and disseminated malignancy. Post-endoscopy diagnosis and major stigmata are added to the pre-endoscopy Rockall score to calculate the complete Rockall score [13]. Meanwhile, the Glasgow–Blatchford score included the heart rate, systolic blood pressure, blood urea nitrogen, and hemoglobin level [14, 15]. The AIMS65 score was calculated by allotting 1 point each for albumin level <30 g/L, INR >1.5, alteration in mental status, systolic blood pressure ≤90 mm Hg, and age ≥65 years [5].

### 2.3. Study Endpoint

The primary endpoint was to evaluate whether the PTAR measured in the ED was an early predictor of ICU admission and mortality for UGIB. If the PTAR was a predictor, the optimal cutoff values that predict ICU admission and mortality would be calculated. The predictive performance was also compared against the traditionally used Rockall score, GBS, and AIMS65 scores.

The indication for ICU admission could not be generalized; therefore, a priority model was used to establish admission criteria [16]. The criteria for ICU admission were the application of a mechanical ventilator, hemodynamic monitoring, or the clinician's decision according to the patient's condition. Patients requiring intensive care and monitoring, receiving ventilator treatment or intravenous cardiovascular medications, with respiratory failure requiring ventilation after surgery, or requiring invasive monitoring were prioritized for ICU admission. Patients who needed immediate treatment at any time during intensive monitoring and those with a chronic disease that could rapidly develop into an internal or surgical disease were secondary priorities. The tertiary priorities were patients with an underlying or acute disease that is difficult to expect and those who require intensive treatment to alleviate acute disease but have limited eligibility for endotracheal intubation or cardiopulmonary resuscitation. The fourth priority is not appropriate for ICU admission [16].

### 2.4. Statistical Analyses

Continuous data are presented as means with standard deviations or medians (interquartile ranges) and analyzed using normality tests. The normality of data distribution was assessed using the Shapiro–Wilk test. Continuous data were analyzed using Student's *t*-test or the Mann–Whitney *U* test, as appropriate. Categorical variables are presented as counts and percentages and were analyzed using the chi-square test or Fisher's exact test, as appropriate. Univariate and multivariate logistic regression analyses were performed to assess the influencing factors of ICU admission and mortality. Significant variables in the univariate logistic regression analysis (those with a *p*-value <0.2) were entered into the multivariable logistic regression analysis. To compare the predictive capability of the PTAR with that of previous risk stratification methods, receiver-operating characteristic curves were created using the optimal cutoff values determined using the Youden Index. All statistical analyses were performed using SPSS ver. 23 (IBM, New York, NY) and MedCalc Statistical Software version 17.5.3 (MedCalc Software, Ostend, Belgium). Statistical significance was set at *p* < 0.05.

## 3. Results

### 3.1. Patient Characteristics

**Figure 1:**
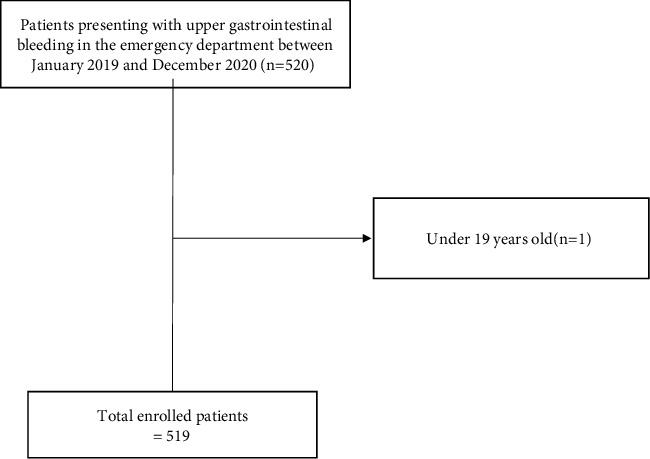
Patient inclusion flowchart.

Among the 520 patients initially identified, a patient who was aged <19 years was excluded (Figure 1). Finally, 519 patients were included in the analysis: 137 (26.4%) had variceal bleeding, 188 (36.2%) had ulcer bleeding, and 194 (37.4%) had other causes of UGIB. Other causes include Mallory-Weiss tear, gastric cancer bleeding, angiodysplasia, and gastric erosion. In this study, all patients with nonvariceal and variceal bleeding were included. Patient characteristics are shown in Table 1.

### 3.2. Comparison between ICU and Non-ICU Patients

In total, 163/519 patients were admitted to the ICU. Compared with the non-ICU group, the ICU group showed significantly lower systolic blood pressure (106 mmHg vs 121 mmHg, *p* ≤ 0.001), hemoglobin count (8.2 g/dL [range, 2.9–18.1 g/dL] vs 10.4 g/dL [range, 3.6–17.5 g/dL], *p* ≤ 0.001), and platelet count (164,000 [range, 19,000–724,000] vs 214,000 [range, 11,000–776,000], *p* ≤ 0.001). Furthermore, the ICU group showed significantly higher levels of blood urea nitrogen (33.9 mg/dL [range, 6.5–138.3 mg/dL] vs 30.1 mg/dL [range, 6.2–184.3 mg/dL], *p* ≤ 0.001) and creatinine (1.18 mg/dL [range, 0.50–10.61 mg/dL] vs 0.96 mg/dL [range, 0.33–19.61 mg/dL], *p* ≤ 0.001). The median PT-INR was higher in the ICU group than in the non-ICU group (1.26 [range, 0.86–5.49] vs 1.08 [0.75–6.63], *p* ≤ 0.001). Meanwhile, the median albumin level was lower in the ICU group (3.1 g/dL [range, 1.1–5.6 g/dL] vs 3.7 g/dL [range, 1.7–5.8 g/dL], *p* ≤ 0.001).

### 3.3. Comparison between Survival Discharge and Mortality

In total, 35 patients died (Table 1). The mortality group showed significantly lower median systolic blood pressure in the ED (100 mmHg [range, 53–254 mmHg] vs. 117 mmHg [range, 53–222 mmHg], *p* ≤ 0.001) and the hemoglobin level (7.7 g/dL [range, 2.9–14.8 g/dL] vs 9.6 g/dL [range, 2.9–18.1 g/dL], *p* ≤ 0.001) and a significantly higher creatinine level (1.5 mg/dL [range, 0.53–10.61 mg/dL] vs 0.98 mg/dL [range, 0.33–19.61 mg/dL], *p* ≤ 0.001). The median PT-INR was higher in the nonsurvivor group than in the survivor group (1.50 [range, 0.95–6.63] vs 1.10 [0.75–4.55], *p* ≤ 0.001). Meanwhile, the nonsurvivor group showed lower albumin levels (*p* ≤ 0.001).

### 3.4. Influencing Factors of ICU Admission

The logistic regression analysis of the factors associated with ICU admission is shown in Table 2. The crude odds ratio (OR) of the PTAR was 40.943 (95% confidence interval [CI], 12.856–130.393), while that of the pre-endoscopy Rockall score, complete Rockall score, GBS, and AIMS65 score was 1.365 (95% CI, 1.194–1.562), 1.390 (95% CI, 1.250–1.546), 1.156 (95% CI, 1.100–1.214), and 1.732 (95% CI, 1.447–2.074), respectively. Univariate logistic regression analysis showed that systolic blood pressure, comorbidities, hemoglobin, and platelet count with a *p*-value <0.2 were significant influencing variables. Thus, these factors, along with age, sex, systolic blood pressure, comorbidities, hemoglobin, and platelet count, were entered into multivariate regression analysis. The adjusted OR (aOR) of the PTAR was 8.376 (2.722–25.774), while the aORs of the complete Rockall score and AIMS65 score were 1.307 (1.102–1.549) and 1.699 (1.318–2.192), respectively. Meanwhile, the aORs of the pre-endoscopy Rockall score and GBS were not significant.

### 3.5. Influencing Factors of Mortality

In logistic regression analysis of influencing factors of mortality (Table 2), the crude OR of the PTAR was 31.525 (11.379–87.340). Meanwhile, the crude ORs of the pre-endoscopy Rockall score, complete Rockall score, GBS, and AIMS65 score were 1.877 (1.421–2.478), 1.631 (1.309–2.031), 1.165 (1.057–1.283), and 2.335 (1.744–3.127), respectively. Univariate logistic regression analysis showed that systolic blood pressure, comorbidities, and hemoglobin were significant factors. Thus, these factors were entered into multivariate regression analysis, along with age, sex, systolic blood pressure, comorbidities, and hemoglobin. The aOR of the PTAR was 27.846 (8.701–89.116). The aOR of the AIMS65 score was 2.154 (1.473–3.149). The aORs of the Rockall score (pre-endoscopy) was 1.647 (1.048–2.589). The aORs of the complete Rockall score and GBS were not significant.

### 3.6. Predictive Factors of ICU Admission and Mortality

Tables 3 and 4 show the optimal cutoff values and diagnostic accuracies of the PTAR, GBS, AIMS65 score, pre-endoscopy Rockall score, and complete Rockall score. Cutoff values of >0.320 (area under the curve [AUC]: 0.720, 95% CI: 0.679–0.758) and >0.358 (AUC: 0.816, 95% CI: 0.780–0.849) for the PTAR were predictive of ICU admission and mortality, respectively. These AUCs were higher than those for other methods of risk stratification. The sensitivity of the PTAR for admission to the ICU was 74.23 (95% CI: 66.8–80.8), and the specificity was 58.43 (95% CI: 53.1–63.6). The sensitivity of the PTAR for mortality was 85.71 (95% CI: 69.7–95.2), and the specificity was 64.88 (95% CI: 60.4–69.1). With regard to AUC values, there was a trend suggesting that PTAR seemed more accurate than the GBS, AIMS65 score, and Rockall score (pre-endoscopy) in ICU admission, and there was a trend suggesting that PTAR seemed more accurate than GBS in terms of mortality (Supplementary Tables F61 and F62).

## 4. Discussion

Current prognostic measures for UGIB are difficult to apply in early ED settings. This study found that the PTAR measured in the ED is an independent factor related to ICU admission and mortality in patients with UGIB. The PTAR had higher predictive capability than the conventional scoring method for predicting prognosis in the ED (i.e., the Rockall score, AIMS65 score, and GBS). An abnormally elevated PTAR was associated with ICU admission and mortality, thus providing a useful marker for the early prediction of prognosis in patients with UGIB.

The Glasgow–Blatchford scoring system, which has been validated as an accurate tool, is one of the most commonly used tools for assessing the severity of early UGIB [14]. The Rockall score, which includes both pre-and post-endoscopic components, has a high predictive capability for mortality [17]. However, because it requires several components, the Rockall score may be challenging to use in the ED [18]. The AIMS65 score was also identified as a good predictive factor for long-term hospitalization and in-hospital mortality. In patients with cirrhosis and UGIB, the AIMS65 score correlated with the length of hospitalization in variceal bleeding [19]. In a previous study, AIMS65, GBS, and Rockall scores were similar in predicting in-hospital mortality (AUC: 0.760 vs 0.780 vs 0.780, respectively) [4]. On the other hand, in a study with a sample size of 424, AIMS65 scores (AUC 0.80) were a better predictor of in-hospital mortality than GBS (AUC 0.76) [3]. In our study, the AUC of AIMS65 was 0.764, higher than the Rockall scores and GBS. In a previous study, the AUC of AIMS65 was higher than GBS and pre-endoscopy Rockall scores, the complete Rockall score for ICU admission (AUC: 0.740 vs 0.700 vs 0.620 vs 0.710) [3]. In our study, the AUC for ICU admission was lower than that reported in previous studies, and the complete Rockall score was higher than the GBS, AIMS65, and pre-endoscopy Rockall scores (AUC 0.670 vs 0.653 vs 0.656 vs 0.617, respectively). This difference may be due to different patient characteristics and ICU admission criteria. Hemoglobin levels have also been used in several studies [13]. The hemoglobin level was linked to favorable outcomes on outpatient follow-up in a previous study [20]. Although the threshold differs among studies, a lower hemoglobin level generally indicates a higher probability of endoscopy before hemodynamic instability and predicts the need for intervention to prevent additional or delayed bleeding [6, 21]. Serum albumin can also be used for risk stratification in patients with UGIB [22, 23].

Low albumin levels may be associated with malnutrition, asymptomatic liver disease, or other chronic diseases; thus, they are also inevitably associated with mortality [23]. A history of any critical disease alters albumin distribution [24], particularly in the rate at which proteins are synthesized and broken down [25]. Coagulopathy frequently occurs in patients with severe disease, and an increase in the PT-INR has been used to predict mortality [26]. Previous studies used the PTAR to evaluate liver function after liver resection in patients with hepatocellular carcinoma [8] and sepsis. The PTAR was also found to have a comparative predictive capability to the Sequential Organ Failure Assessment score for critically ill patients [10]. Another study also reported the usefulness of the PTAR for evaluating the prognosis and 30-day adverse outcomes in patients with liver disease [27].

To the best of our knowledge, our study is the first to investigate the usefulness of the PTAR in predicting the prognosis of patients with UGIB. Explanatory power in ICU admission was higher than that in the other scoring systems. The AUC for mortality was 0.816, which was also higher than that of the other scoring systems. Based on previous findings that the PTAR is related to liver disease, the PTAR can be easily used to predict the prognosis of UGIB patients in the emergency department. However, this study also has some limitations. First, some data may have been omitted because of the retrospective nature of the study. Patients with incomplete laboratory tests were excluded in this study, and patients on warfarin and those with known cirrhosis were not included in the studied subjects, which is a limitation of the retrospective study and is likely to affect the results. Second, the sample size was small because this study was conducted at the ED of a single university tertiary hospital. Third, the time from the symptom onset to ED arrival may affect the PTAR value, but this was not evaluated. Fourth, the MELD score and Child-Pugh score used in patients with liver cirrhosis and variceal bleeding were not calculated or evaluated. Fifth, indications for ICU admission may vary depending on the institution, meaning that predictions for ICU admission may be different among hospitals, depending on their clinical capability and resources. Finally, the rebleeding risk was not evaluated. Therefore, these limitations may be considered for future studies, including the validation process.

## 5. Conclusions

The PTAR measured in the ED is an independent factor related to ICU admission and mortality in patients with UGIB. Thus, the PTAR can be applied as a risk stratification marker for UGIB in the early emergency setting.

## Figures and Tables

**Table 1:** Patient characteristics.

	All patients *n* = 519	Non-ICU *n* = 356	ICU^,^*n* = 163	*p*-Value	Survival discharge *n* = 484	Mortality *n* = 35	*p*-Value
Age (years)	63.86 ± 17.04	63.84 ± 18.31	63.91 ± 13.93	0.533	63.49 ± 17.20	69.03 ± 13.84	0.086
Male, *n* (%)	343 (66.1%)	216 (60.7%)	127 (77.9%)	≤0.001	315 (65.1%)	28 (80%)	0.106
SBP, mmHg	116 (53–254)	121 (53–222)	106 (53–254)	≤0.001	117 (53–222)	100 (53–254)	≤0.001
Pulse, beats/min	97 (40–193)	94 (44–166)	98 (40–193)	0.122	97 (40–193)	96 (43–146)	0.556
Comorbidities	450 (86.7%)	296 (83.1%)	154 (94.5%)	0.001	415 (85.7%)	35 (100%)	0.032
Hemoglobin, g/dL	9.6 (2.9–18.1)	10.4 (3.6–17.5)	8.2 (2.9–18.1)	≤0.001	9.6 (2.9–18.1)	7.7 (2.9–14.8)	≤0.001
Platelet, ×E^9^/L	205 (11–776)	214 (11–776)	164 (19–724)	≤0.001	206 (11–776)	151 (40–440)	0.030
BUN, mg/dL	31.2 (6.2–184.3)	30.1 (6.2–184.3)	33.9 (6.5–138.3)	0.022	30.8 (6.3–184.3)	38.6 (6.5–151.9)	0.034
Creatinine, mg/dL	0.99 (0.33–19.61)	0.96 (0.33–19.61)	1.18 (0.50–10.61)	≤0.001	0.98 (0.33–19.61)	1.5 (0.53 10.61)	≤0.001
PT-INR	1.11 (0.75–6.63)	1.08 (0.75–6.63)	1.26 (0.86–5.49)	≤0.001	1.10 (0.75–4.55)	1.50 (0.95–6.63)	≤0.001
Albumin, g/dL	3.5 (1.1–5.8)	3.7 (1.7–5.8)	3.1 (1.1–5.6)	≤0.001	3.6 (1.4–5.8)	2.9 (1.1–5.1)	≤0.001

ICU, intensive care unit; SBP, systolic blood pressure; BUN, blood urea nitrogen; PT-INR, prothrombin time-international normalized ratio.

**Table 2:** Influencing factors of ICU admission and mortality.

Factor	Non-ICU “(*n* = 356)	ICU “(*n* = 163)	ICU admission		Survival “(*n*=484)	Mortality “(*n*=35)	Mortality	
			Crude OR “(95% CI)	Adjusted OR^a^ “(95% CI)			Crude OR “(95% CI)	Adjusted OR^b^ “(95% CI)
PTAR	0.293 (0.153–1.745)	0.390 (0.188– 1.685)	40.943 (12.856–130.393)	8.376 2.722–25.774	0.319 (0.153–1.685)	0.574 (0.206–1.745)	31.525 (11.379–87.340)	27.846 (8.701–89.116)
GBS	8 (0–16)	10 (0–16)	1.156 (1.100–1.214)	0.991 0.906–1.085	9 (0–16)	11 (3–16)	1.165 (1.057–1.283)	0.963 (0.822–1.127)
AIMS65 score	1 (0–5)	2 (0–5)	1.732 (1.447–2.074)	1.699 1.318–2.192	1 (0–5)	2 (0–5)	2.335 (1.744–3.127)	2.154 (1.473–3.149)
Rockall score (pre-endoscopy)	4 (0–7)	4 (0–7)	1.365 (1.194–1.562)	1.238 0.957–1.601	4 (0–7)	5 (2–7)	1.877 (1.421–2.478)	1.647 (1.048–2.589)
Rockall score (complete)	6 (0–10)	7 (3–10)	1.390 (1.250–1.546)	1.307 1.102–1.549	6 (0–10)	7 (4–10)	1.631 (1.309–2.031)	1.363 (0.999–1.858)

ICU, intensive care unit; OR, odds ratio; CI, confidence interval; PTAR, prothrombin time-international normalized ratio-to-albumin ratio; GBS, Glasgow–Blatchford Score; AIM65, albumin, international normalized ratio, mental status, systolic blood pressure, age>65 years. ^a^Controlled for age, sex, systolic blood pressure, comorbidities, hemoglobin, and platelet count. ^b^Controlled for age, sex, systolic blood pressure, comorbidities, and hemoglobin.

**Table 3:** Predictive variables of ICU admission.

Variables	AUC (95% CI)	Cutoff	Sensitivity (95% CI)	Specificity (95% CI)	PPV (95% CI)	NPV (95% CI)
PTAR	0.720 (0.679–0.758)	>0.320	74.23 (66.8–80.8)	58.43 (53.1–63.6)	45.0 (41.2–48.8)	83.2 (79.0–86.7)
GBS	0.653 (0.610–0.694)	>6	84.66 (78.2–89.8)	41.01 (35.9–46.3)	39.7 (37.1–42.3)	85.4 (79.9–89.5)
AIMS65 score	0.656 (0.613–0.697)	>1	51.53 (43.6–59.4)	75.56 (70.8–79.9)	49.1 (43.3–55.0)	77.3 (74.2–80.1)
Rockall score (pre-endoscopy)	0.617 (0.574–0.659)	>4	39.26 (31.7–47.2)	78.09 (73.4–82.3)	45.1 (38.4–51.9)	73.7 (71.0–76.3)
Rockall score (complete)	0.670 (0.628–0.710)	>5	76.69 (69.4–82.9)	49.44 (44.1–54.8)	41 (37.8–44.2)	82.2 (77.5–86.2)

ICU, intensive care unit; AUC, area under the curve; CI, confidence interval; PPV, positive predictive value; NPV, negative predictive value; PTAR, prothrombin time-international normalized ratio-to-albumin ratio; GBS, Glasgow–Blatchford Score; AIM65, albumin, international normalized ratio, mental status, systolic blood pressure, age >65 years.

**Table 4:** Predictive variables of mortality.

Variables	AUC (95% CI)	Cutoff	Sensitivity (95% CI)	Specificity (95% CI)	PPV (95% CI)	NPV (95% CI)
PTAR	0.816 (0.780–0.849)	>0.358	85.71 (69.7–95.2)	64.88 (60.4–69.1)	15.0 (12.8–17.5)	98.4 (96.5–99.3)
GBS	0.657 (0.614–0.697)	>5	94.29 (80.8–99.3)	29.55 (25.5–33.8)	8.8 (8.1–9.7)	98.6 (94.9–99.6)
AIMS65 score	0.764 (0.725–0.800)	>1	68.57 (50.7–83.1)	69.63 (65.3–73.7)	14.0 (11.2–17.5)	96.8 (94.9–98.0)
Rockall score (pre-endoscopy)	0.736 (0.696–0.773)	>3	94.29 (80.8–99.3)	43.39 (38.9–47.9)	10.7 (9.7–11.9)	99.1 (96.5–99.8)
Rockall score (complete)	0.741 (0.701–0.778)	>6	82.86 (66.4–93.4)	61.78 (57.3–66.1)	13.6 (11.5–15.9)	98.0 (96.0–99.0)

AUC, area under the curve; CI, confidence interval; PPV, positive predictive value; NPV, negative predictive value; PTAR, prothrombin time-international normalized ratio-to-albumin ratio; GBS, Glasgow–Blatchford score; AIM65, albumin, international normalized ratio, mental status, systolic blood pressure, age >65 years.

## Data Availability

Data cannot be shared to the public because of institutional and national data policy restrictions. Data can be made available to researchers who meet the criteria for access to confidential data.

## Supplementary Materials

Supplementary Materials Supplement Table 1. Pairwise comparison of receiver-operating characteristic (ROC) curves for ICU admission. Supplement Table 2. Pairwise comparison of receiver-operating characteristic (ROC) curves for mortality
