# Supplementary material for: International Normalized Ratio-to-Albumin Ratio as a Novel Marker of Upper Gastrointestinal Bleeding Severity
Source: Gastroenterol Res Pract. 2022 Oct 13;2022:1172540. doi: 10.1155/2022/1172540 (PMC9584709; doi:10.1155/2022/1172540)
Supplement: Supplementary Materials — Supplement Table 1. Pairwise comparison of receiver-operating characteristic (ROC) curves for ICU admission. Supplement Table 2. Pairwise comparison of receiver-operating characteristic (ROC) curves for mortality [file 1172540.f1.docx]

Supplement Table 1.

Pairwise comparison of receiver-operating characteristic (ROC) curves for ICU admission

| Variables | GBS | AIMS65 score | Rockall score (Pre-endoscopy) | Rockall score (Complete) |
| --- | --- | --- | --- | --- |
| PTAR |  |  |  |  |
| Difference between areas | 0.0675 | 0.0645 | 0.103 | 0.0503 |
| Standard Error ^a^ | 0.0247 | 0.0244 | 0.0290 | 0.0273 |
| z statistic | 2.729 | 2.643 | 3.551 | 1.842 |
| p-value | 0.0063 | 0.0082 | 0.0004 | 0.0655 |
| GBS |  |  |  |  |
| Difference between areas |  | 0.00307 | 0.0356 | 0.0173 |
| Standard Error ^a^ |  | 0.0285 | 0.0267 | 0.0255 |
| z statistic |  | 0.108 | 1.330 | 0.677 |
| p-value |  | 0.9142 | 0.1834 | 0.4984 |
| AIMS65 score |  |  |  |  |
| Difference between areas |  |  | 0.0386 | 0.0142 |
| Standard Error ^a^ |  |  | 0.0242 | 0.0260 |
| z statistic |  |  | 1.599 | 0.546 |
| p-value |  |  | 0.1099 | 0. 5853 |
| Rockall score (Pre-endoscopy) |  |  |  |  |
| Difference between areas |  |  |  | 0.0528 |
| Standard Error ^a^ |  |  |  | 0.0152 |
| z statistic |  |  |  | 3.469 |
| p-value |  |  |  | 0.0005 |

^a^ Hanley & McNeil, 1983, ICU, intensive care unit; CI, confidence interval; PTAR, prothrombin time-international normalized ratio-to-albumin ratio; GBS, Glasgow–Blatchford score; AIM65, albumin, international normalized ratio, mental status, systolic blood pressure, age >65 years

Supplement Table 2.

Pairwise comparison of Receiver-Operating Characteristic (ROC) curves for mortality

| Variables | GBS | AIMS65 score | Rockall score (Pre-endoscopy) | Rockall score (Complete) |
| --- | --- | --- | --- | --- |
| PTAR |  |  |  |  |
| Difference between areas | 0.160 | 0.0528 | 0.0805 | 0.0757 |
| Standard Error ^a^ | 0.0468 | 0.0356 | 0.0432 | 0.0432 |
| z statistic | 3.415 | 1.484 | 1.860 | 1.752 |
| p-value | 0.0006 | 0.1379 | 0.0628 | 0.0798 |
| GBS |  |  |  |  |
| Difference between areas |  | 0.107 | 0.0795 | 0.0842 |
| Standard Error ^a^ |  | 0.0482 | 0.0463 | 0.0473 |
| z statistic |  | 2.224 | 1.715 | 1.782 |
| p-value |  | 0.0261 | 0.0863 | 0.0748 |
| AIMS65 score |  |  |  |  |
| Difference between areas |  |  | 0.0277 | 0.0229 |
| Standard Error ^a^ |  |  | 0.0346 | 0.0382 |
| z statistic |  |  | 0.800 | 0.599 |
| p-value |  |  | 0.4239 | 0.5489 |
| Rockall score (Pre-endoscopy) |  |  |  |  |
| Difference between areas |  |  |  | 0.00475 |
| Standard Error ^a^ |  |  |  | 0.0261 |
| z statistic |  |  |  | 0.182 |
| p-value |  |  |  | 0.8558 |

^a^ Hanley & McNeil, 1983, CI, confidence interval; PTAR, prothrombin time-international normalized ratio-to-albumin ratio; GBS, Glasgow–Blatchford score; AIM65, albumin, international normalized ratio, mental status, systolic blood pressure, age >65 years
